# Soft Quantization Using Entropic Regularization

**DOI:** 10.3390/e25101435

**Published:** 2023-10-10

**Authors:** Rajmadan Lakshmanan, Alois Pichler

**Affiliations:** Faculty of Mathematics, Technische Universität Chemnitz, D-09111 Chemnitz, Germany; alois.pichler@math.tu-chemnitz.de

**Keywords:** quantization, approximation of measures, entropic regularization, 94A17, 81S20, 40A25

## Abstract

The quantization problem aims to find the best possible approximation of probability measures on $R^{d}$ using finite and discrete measures. The Wasserstein distance is a typical choice to measure the quality of the approximation. This contribution investigates the properties and robustness of the entropy-regularized quantization problem, which relaxes the standard quantization problem. The proposed approximation technique naturally adopts the softmin function, which is well known for its robustness from both theoretical and practicability standpoints. Moreover, we use the entropy-regularized Wasserstein distance to evaluate the quality of the soft quantization problem’s approximation, and we implement a stochastic gradient approach to achieve the optimal solutions. The control parameter in our proposed method allows for the adjustment of the optimization problem’s difficulty level, providing significant advantages when dealing with exceptionally challenging problems of interest. As well, this contribution empirically illustrates the performance of the method in various expositions.

## 1. Introduction

Over the past few decades, extensive research has been conducted on optimal quantization techniques in order to tackle numerical problems that are related to various fields such as data science, applied disciplines, and economic models. These problems are typically centered around *uncertainties* or *probabilities* which demand robust and efficient solutions (cf. Graf and Mauldin [1], Luschgy and Pagès [2], El Nmeir et al. [3]). In general, these problems are difficult to handle, as the random components in the problem allow uncountably many outcomes. As a consequence, in order to address this difficulty the probability measures are replaced by simpler or finite measures, which can facilitate numerical computations. However, the probability measures should be ‘close’ in order to ensure that the result of the computations with approximate (discrete) measures resembles the original problem. In a nutshell, the goal is to find the best approximation of a diffuse measure using a discrete measure, which is called an *optimal quantization* problem. For a comprehensive discussion of the optimal quantization problem from a mathematical standpoint, refer to Graf and Luschgy [4].

On the other hand, *entropy* (sometimes known as information entropy) is an essential concept when dealing with uncertainties and probabilities. In mathematics, entropy is often used as a measure of information and uncertainty. It provides a quantitative measure of the randomness or disorder in a system or a random variable. Its applications span information theory, statistical analysis, probability theory, and the study of complex dynamical systems (cf. Breuer and Csiszár [5,6], Pichler and Schlotter [7]).

In order to assess the closeness of probability measures, distances are often considered; one notable instance is the Wasserstein distance. Ostensibly, the Wasserstein distance measures the minimum average amount of transporting cost required to transfer one probability measure into another. Unlike other formulations of distances and/or divergence, which simply compare the probabilities of the distribution functions (e.g., the total variation distance and the Kullback–Leibler divergence), the Wasserstein distance incorporates the geometry of the underlying space. This increases the understanding of the relationships between different probability measures in a geometrically trustworthy manner.

In our research work, we focus on entropy-regularized quantization methods. More precisely, we consider an entropy-regularized version of the Wasserstein problem to quantify the quality of the approximation, and adapt the stochastic gradient approach to obtain the optimal quantizers.

The key features of our methodology include the following:

(i) Our regularization approach stabilizes and simplifies the standard quantization problem by introducing penalty terms or constraints that discourage overly complex or overfitted models, promoting better generalizations and robustness in the solutions.
(ii) The influence of entropy is controlled using a parameter, λ, which enables us to reach the genuine optimal quantizers.
(iii) Generally, parameter tuning comes with certain limitations. However, our method builds upon the framework of the well-established softmin function, which allows us to exercise parameter control without encountering any restrictions.
(iv) For larger values of the regularization parameter λ, the optimal measure accumulates all its mass at the center of the measure.

**Applications in the Context of Quantization.**

Quantization techniques have undergone significant developments in recent years, particularly within the domain of deep learning and model optimization. State-of-the-art research has introduced advanced methodologies such as non-uniform quantization and quantization-aware training, enabling the efficient deployment of neural networks while preserving performance (cf. Jacob et al. [8], Zhuang et al. [9], Hubara et al. [10]). Furthermore, quantization principles have found applications beyond machine learning, such as in digital image processing, computer vision (cf. Polino et al. [11]), and electric charge quantization (cf. Bhattacharya [12]).

**Related Works and Contributions.**

As mentioned above, optimal quantization is a well-researched topic in the field of information theory and signal processing. Several methods have been developed for the optimal quantization problem, notably including the following:

– Lloyd-Max Algorithm: this algorithm, also known as Lloyd’s algorithm or the *k*-means algorithm, is a popular iterative algorithm for computing optimal vector quantizers. It iteratively adjusts the centroids of the quantization levels to minimize the quantization error (cf. Scheunders [13]).
– Tree-Structured Vector Quantization (TSVQ): TSVQ is a hierarchical quantization method that uses a tree structure to partition the input space into regions. It recursively applies vector quantization at each level of the tree until the desired number of quantization levels is achieved (cf. Wei and Levoy [14]).
– Expectation-maximization (EM) algorithm: the EM algorithm is a general-purpose optimization algorithm that can be used for optimal quantization. It is an iterative algorithm that estimates the parameters of a statistical model to maximize the likelihood of the observed data (cf. Heskes [15]).
– Stochastic Optimization Methods: stochastic optimization methods such as simulated annealing, genetic algorithms, and particle swarm optimization can be used to find optimal quantization strategies by exploring the search space and iteratively improving the quantization performance (cf. Pagès et al. [16]).
– Greedy vector quantization (GVQ): the greedy algorithm tries to solve this problem iteratively by adding one code word at every step until the desired number of code words is reached, each time selecting the code word that minimizes the error. GVQ is known to provide suboptimal quantization compared to other non-greedy methods such as the Lloyd-Max and Linde–Buzo–Gray algorithms. However, it has been shown to perform well when the data have a strong correlation structure. Notably, it utilizes the Wasserstein distance to measure the error of approximation (cf. Luschgy and Pagès [2]).

These methods provide efficient and practical solutions for finding optimal quantization schemes, and have different trade-offs between complexity and performance. The choice of method depends on the problem of interest and the requirements of the application. However, most of these methods depend on strict constraints, which makes the solutions overly complex or results in model overfitting. Our method mitigates this issue by promoting better generalizations and robustness in the solutions.

In the optimal transport community, the entropy-regularized version of the optimal transport problem (known as the entropy-regularized Wasserstein problem) was initial proposed in Cuturi [17]. This entropy version of the Wasserstein problem promotes fast computations using Sinkhorn’s algorithm. As an avenue for constructive research, the above-cited study presented a multitude of results aimed at gaining a comprehensive understanding of the subtleties involved in enhancing the computational performance of entropy-optimal transport (cf. Ramdas et al. [18], Neumayer and Steidl [19], Altschuler et al. [20], Lakshmanan et al. [21], Ba and Quellmalz [22], Lakshmanan and Pichler [23]). These findings have served as a valuable foundation for further exploration in the field of optimal transport, providing insights into both the intricacies of the topic and potential avenues for improvement.

In contrast, we present a new and innovative approach that concentrates on the optimal quantization problem based on entropy and on its robust properties, which represents a distinct contribution with regard to standard entropy-regularized optimal transport problems.

One of the principal consequences of our research substantiates the convergence behavior of quantizers at the center of the measure. The relationship between the center of the measure and the entropy-regularized quantization problem has not been exposed yet. The following plain solution is obtained by intensifying the entropy term in the regularization of the quantization problem.

**Theorem** **1.** *There exists a real valued $\lambda_{0}>0$ such that the approximation of the entropy-regularized optimal quantization problem is provided by the Dirac measure*

$$P=\delta_{a}$$

*for every $\lambda >\lambda_{0}$, where a is the center of the measure P with respect to the distance d.*

This enthralling interpretation (Theorem 1) of our master problem facilitates an understanding of the transition from a complex and difficult optimization solution to a simple solution. Moreover, along with a theoretical discussion, we provide an algorithm and numerical exemplification which empirically demonstrate the robustness of our method. The forthcoming sections elucidate the robustness and asymptotic properties of the proposed method in detail.

**Outline of the Paper.**

Section 2 establishes the essential notation, definitions, and properties. Moreover, we comprehensively expound upon the significance of the smooth minimum, a pivotal component in our research. In Section 3, we introduce the entropy-regularized optimal quantization problem and delve into its inherent properties. Section 4 presents a discussion of the soft tessellation, optimal weights, and theoretical properties of parameter tuning. Furthermore, we systematically illustrate the computational process along with a pseudo-algorithm. Section 5 provides numerical examples and empirically substantiates the theoretical proofs. Finally, Section 6 summarizes the study.

## 2. Preliminaries

In what follows, (X,d) is a Polish space. The σ-algebra generated by the Borel sets induced by the distance *d* is F, while the set of all probability measures on X is P(X).

### 2.1. Distances and Divergences of Measures

The standard quantization problem employs the Wasserstein distance to measure the quality of the approximation, which was initially studied by Monge and Kantorovich (cf. Monge [24], Kantorovich [25]). One of the remarkable properties of this distance is that it metrizes the weak* topology of measures.

**Definition** **1** (Wasserstein distance)**.** *Let P and $\tilde{P}$ be probability measures on (X,d). The Wasserstein distance of order r≥1 between P and $\tilde{P}\in P\left(X\right)$ is*

(1) $$d_{r}\left(P,\tilde{P}\right):=\inf{\left(\underset{X\times X}{\;\int \int \;}d{\left(\xi ,\tilde{\xi }\right)}^{r}\;\pi \left(d\xi ,d\tilde{\xi }\right)\right)}^{1/r},$$

*where the infimum is among all measures $\pi \in P(X^{2})$ with marginals P and $\tilde{P}$, that is,*

(2) $$\begin{array}{cc} \pi (A\times X) & =P(A)\;and\; \end{array}$$

(3) $$\begin{array}{cc} \pi (X\times B) & =\tilde{P}\left(B\right) \end{array}$$

*for all sets A and B∈F. The measures*

$$\pi_{1}\left(\cdot \right):=\pi \left(\cdot \times X\right)\;\;and\;\;\pi_{2}\left(\cdot \right):=\pi \left(X\times \cdot \right)$$

*on X are called the *marginal measures* of the bivariate measure π.*

Readers may refer to the excellent monographs in [26,27] for a comprehensive discussion of the Wasserstein distance.

**Remark** **1** (Flexibility)**.** *In the subsequent discussion, our problem of interest is to approximate the measure P, which is a continuous, discrete, or mixed measure on $X=R^{d}$. The measure $\tilde{P}$ is used to approximate the measure P, which is a discrete measure. The definition of the Wasserstein distance flexibly comprises all the cases, namely, continuous, semi-discrete, and discrete measures.*

In contrast to the standard methodology, we investigate the quantization problem by utilizing an entropy version of the Wasserstein distance. The standard Wasserstein problem is regularized by adding the Kullback–Leibler divergence, which is known as the relative entropy.

**Definition** **2** (Kullback–Leibler divergence)**.** *Let P and Q∈P(X) be probability measures. Denote the Radon–Nikodým derivative dQ=Z dP by $Z\in L^{1}\left(P\right)$ if Q is absolutely continuous with respect to P (Q≪P). The *Kullback–Leibler divergence *is*

(4) $$D\left(Q∥P\right):=\left\{\begin{array}{cc} E_{P}Z\log Z=E_{Q}\log Z & if\;Q\ll P\;and\;dQ=Z\;dP,\\ +\infty & else, \end{array}\right.$$

*where $E_{P}$ ($E_{Q}$, resp.) is the expectation with respect to the measure P (Q, resp.).*

Per Gibbs’ inequality, the Kullback–Leibler divergence satisfies D(Q∥P)≥0 (non-negativity). However, *D* is not a distance metric, as it does not satisfy the symmetry and triangle inequality properties.

We would like to emphasize the following distinctness with respect to the Wasserstein distance (cf. Remark 1): in order for the Kullback–Leibler divergence to be finite (D(Q∥P)<∞), we must have

suppQ⊂suppP,

where the support of the measure (cf. Rüschendorf [28]) is

$$\mathrm{supp}\;P:=⋂\left\{A\in F:A\;\mathrm{is}\;\mathrm{closed}\;\mathrm{and}\;P(A)=1\right\}.$$

If *P* is a continuous measure on $X=R^{d}$, then *Q* is as well. If *P* is a finite measure, then the support points of *P* contain the support points of *Q*.

### 2.2. The Smooth Minimum

In what follows, we present the smooth minimum in its general form, which includes discrete and continuous measures. The numerical computations in the following section rely on results for its discrete version. Therefore, we address the special properties of its discrete version in detail.

**Definition** **3** (Smooth minimum)**.** *Let λ>0 and let Y be a random variable. The *smooth minimum*, or *smooth minimum with respect to $\tilde{P}$*, is*

(5) $$\begin{array}{cc} \min_{\tilde{P};\;\lambda }(Y) & :=-\lambda \log E_{\tilde{P}}e^{-Y/\lambda } \end{array}$$

(6) $$\begin{array}{cc} \; & =-\lambda \log\int_{X}e^{-Y(\eta )/\lambda }\;\tilde{P}\left(d\eta \right), \end{array}$$

*provided that the expectation (integral) of $e^{-Y/\lambda }$ is finite, or if it is not finite, that $\min_{\tilde{P};\;\lambda }(Y):=-\infty$. For λ=0, we set*

(7) $$\min_{\tilde{P};\;\lambda =0}(Y):=ess infY.$$

*For a σ-algebra G⊂F and λ>0 measurable with respect to G, the *conditional smooth minimum *is*

$$\min_{\tilde{P};\lambda }\left(Y|\;G\right):=-\lambda \log E_{\tilde{P}}\left(\left.e^{-Y/\lambda }\right|\;G\right).$$

The following lemma relates the smooth minimum with the essential infimum (cf. (7)), that is, colloquially, the ‘minimum’ of a random variable. As well, the result justifies the term *smooth minimum*.

**Lemma** **1.** *For λ>0, it holds that*

(8) $$\min_{\tilde{P};\lambda }\left(Y\right)\leq E_{\tilde{P}}Y$$

*and*

(9) $$ess infY\leq \min_{\tilde{P};\lambda }\left(Y\right)\underset{\lambda \to 0}{\to }ess infY.$$

**Proof.**  Inequality (8) follows from Jensen’s inequality as applied to the convex function x↦exp(−x/λ).

Next, the first inequality in the second display (9) follows from ess infY≤Y and the fact that all operations in (6) are monotonic. Finally, let a>ess infY. Per Markov’s inequality, we have

(10) $$E_{\tilde{P}}e^{-Y/\lambda }\geq e^{-a/\lambda }\;\tilde{P}\left(e^{-Y/\lambda }\geq e^{-a/\lambda }\right)=e^{-a/\lambda }\;\tilde{P}\left(Y\leq a\right),$$

which is a variant of the Chernoff bound. From Inequality (10), it follows that

(11) $$\min_{\tilde{P};\lambda }\left(Y\right)=-\lambda \log E_{\tilde{P}}e^{-Y/\lambda }\leq -\lambda \log\left(e^{-a/\lambda }\;\tilde{P}\left(Y\leq a\right)\right)=a+\lambda \log\frac{1}{\tilde{P}\left(Y\leq a\right)}.$$

When λ>0 and λ→0, we have

$$\min_{\tilde{P};\lambda }(Y)\leq a,$$

where *a* is an arbitrary number with a>ess infY. This completes the proof.    □

**Remark** **2** (Nesting property)**.** *The main properties of the smooth minimum include translation equivariance*

$$\min_{\tilde{P};\lambda }(Y+c)=\min_{\tilde{P};\lambda }(Y)+c,\;c\in R,$$

*and positive homogeneity*

$$\min_{\tilde{P};\gamma \cdot \lambda }(\gamma \cdot Y)=\gamma \cdot \min_{\tilde{P};\lambda }(Y),\;\gamma >0.$$

*As a consequence of the tower property of the expectation, we have the nesting property*

$$\min_{\tilde{P};\lambda }\left(\min_{\tilde{P};\lambda }(Y|\;G)\right)=\min_{\tilde{P};\lambda }\left(Y\right),$$

*provided that G is a sub-σ-algebra of F.*

### 2.3. Softmin Function

The smooth minimum is related to the softmin function via its derivatives. In what follows, we express variants of its derivatives, which are involved later.

**Definition** **4** (Softmin function)**.** *For λ>0 and a random variable Y with a finite smooth minimum, the *softmin function* is the random variable*

(12) $$\sigma_{\lambda }\left(Y\right):=\exp\left(-\frac{Y-\min_{\tilde{P};\lambda }(Y)}{\lambda }\right)=\frac{e^{-Y/\lambda }}{E_{\tilde{P}}e^{-Y/\lambda }},$$

*where the latter equality is obvious based on the definition of the smooth minimum in *(6)*. The function $\sigma_{\lambda }\left(Y\right)$ is called the *Gibbs density.

**The Derivative with respect to the Probability Measure**

The definition of the smooth minimum in (6) does not require the measure $\tilde{P}$ to be a probability measure. Based on $\frac{\partial }{\partial t}\log\left(a+t\cdot h\right)=\frac{h}{a}$ (at t=0) for the natural logarithm, the directional derivative of the smooth minimum in the direction of the measure *Q* is

(13) $$\begin{array}{c} \frac{1}{t}\left(\min_{\tilde{P}+t\cdot Q;\lambda }(Y)-\min_{\tilde{P};\lambda }(Y)\right) \end{array}$$

(14) $$\begin{array}{cc} \; & =-\frac{\lambda }{t}\left(\log\int_{X}e^{-Y/\lambda }\;d\left(\tilde{P}+t\cdot Q\right)-\log\int_{X}e^{-Y/\lambda }\;d\tilde{P}\right) \end{array}$$

(15) $$\begin{array}{cc} \; & \underset{t\to 0}{\to }-\lambda \cdot \frac{\int_{X}e^{-Y/\lambda }\;dQ}{\int_{X}e^{-Y/\lambda }\;d\tilde{P}} \end{array}$$

(16) $$\begin{array}{cc} \; & =-\lambda \cdot \int_{X}\sigma_{\lambda }\left(Y\right)\;dQ. \end{array}$$

Note that $-\lambda \;\sigma_{\lambda }$ is (up to the constant −λ) a Radon–Nikodým density in (16). Thus, the Gibbs density $\sigma_{\lambda }\left(Y\right)$ is proportional to the directional derivative of the smooth minimum with respect to the underlying measure $\tilde{P}$.

**The Derivative with respect to the Random Variable**

In what follows, we additionally require the derivative of the smooth minimum with respect to its argument. Following similar reasoning as above, this is accomplished by

(17) $$\begin{array}{c} \frac{1}{t}\left(\min_{\tilde{P};\lambda }(Y+t\cdot Z)-\min_{\tilde{P};\lambda }(Y)\right) \end{array}$$

(18) $$\begin{array}{cc} \; & =-\frac{\lambda }{t}\left(\log\int_{X}e^{-(Y+t\cdot Z)/\lambda }\;d\tilde{P}-\log\int_{X}e^{-Y/\lambda }\;d\tilde{P}\right) \end{array}$$

(19) $$\begin{array}{cc} \; & =-\frac{\lambda }{t}\left(\log\int_{X}e^{-Y/\lambda }\left(1-\frac{t}{\lambda }Z+𝒪\left(t^{2}\right)\right)\;d\tilde{P}-\log\int_{X}e^{-Y/\lambda }\;d\tilde{P}\right) \end{array}$$

(20) $$\begin{array}{cc} \; & \underset{t\to 0}{\to }\frac{\int_{X}Z\cdot e^{-Y/\lambda }\;d\tilde{P}}{\int_{X}e^{-Y/\lambda }\;d\tilde{P}} \end{array}$$

(21) $$\begin{array}{cc} \; & =\int_{X}Z\cdot \sigma_{\lambda }\left(Y\right)\;d\tilde{P}, \end{array}$$

which involves the softmin function $\sigma_{\lambda }\left(\cdot \right)$ as well.

## 3. Regularized Quantization

This section introduces the entropy-regularized optimal quantization problem along with its properties; we first recall the standard optimal quantization problem.

The standard quantization measures the quality of the approximation using the Wasserstein distance and considers the following problem (cf. Graf and Luschgy [4]):

(22) $$\underset{\pi :\begin{array}{c} \pi_{1}=P,\;\\ \pi_{2}\in P_{m}\left(X\right) \end{array}}{\inf}\underset{X\times X}{\;\int \int \;}d\left(\xi ,\tilde{\xi }\right)\;\pi \left(d\xi ,d\tilde{\xi }\right),$$

where

(23) $$P_{m}\left(X\right):=\left\{{\tilde{P}}_{m}\in P\left(X\right):{\tilde{P}}_{m}=\sum_{j=1}^{m}{\tilde{p}}_{j}\;\delta_{y_{j}}\right\}$$

is the set of measures on X supported by not more than *m* (m∈N) points.

Soft quantization (or quantization regularized with the Kullback–Leibler divergence) involves the regularized Wasserstein distance instead of (22). The soft quantization problem is regularized with the Kullback–Leibler divergence:

(24) $$\inf\left\{E_{\pi }d^{r}+\lambda \cdot D\left(\pi ∥\;P\times {\tilde{P}}_{m}\right):\pi_{1}=P\;\mathrm{and}\;\pi_{2}={\tilde{P}}_{m}\in P_{m}\left(X\right)\right\},$$

where λ>0 and $E_{\pi }d^{r}={\;\int \int \;}_{X^{2}}d{\left(\xi ,\tilde{\xi }\right)}^{r}\;\pi \left(d\xi ,d\tilde{\xi }\right)$. The optimal measure ${\tilde{P}}_{m}\in P_{m}\left(X\right)$ for solving (24) depends on the regularization parameter λ.

In the following discussion, we initially investigate the regularized approximation, which again demonstrates the existence of an optimal approximation.

### 3.1. Approximation with Inflexible Marginal Measures

The following proposition addresses the optimal approximation problem after being regularized with the Kullback–Leibler divergence and fixed marginals. To this end, we dissect the infimum in the soft quantization problem (24) as follows:

(25) $$\underset{{\tilde{P}}_{m}\in P_{m}\left(X\right)}{\inf}\underset{\pi :\begin{array}{c} \pi_{1}=P,\\ \pi_{2}={\tilde{P}}_{m} \end{array}}{\inf}E_{\pi }d^{r}+\lambda \cdot D\left(\pi ∥\;P\times {\tilde{P}}_{m}\right),$$

where the marginals *P* and ${\tilde{P}}_{m}$ are fixed in the inner infimum.

The following Proposition 1 addresses this problem with a fixed bivariate distribution, which is the inner infimum in (25). Then, Proposition 2 reveals that the optimal marginals coincide in this case.

**Proposition** **1.** *Let P be a probability measure and let λ>0. The inner optimization problem in *(25)* relative to the fixed bivariate distribution $P\times \tilde{P}$ is provided by the explicit formula*

(26) $$\begin{array}{cc} \underset{\pi :\pi_{1}=P}{\inf}E_{\pi }d^{r}+\lambda \cdot D\left(\pi ∥P\times \tilde{P}\right)\; & =-\lambda \int_{X}\log\int_{X}e^{-d{\left(\xi ,\tilde{\xi }\right)}^{r}/\lambda }\;\tilde{P}\left(d\tilde{\xi }\right)\;P\left(d\xi \right) \end{array}$$

(27) $$\begin{array}{cc} \; & =E_{\xi ∼P}\left(\min_{\tilde{\xi }∼\tilde{P};\lambda }d{\left(\xi ,\tilde{\xi }\right)}^{r}\right), \end{array}$$

*where $D(\pi ∥P\times \tilde{P})$ is the Kullback–Leibler divergence. Further, the infimum in *(26)* is attained.*

**Remark** **3.** *The notation in *(27)* (*(29)* below, resp.) is chosen to reflect the explicit expression in *(26)*, while the soft minimum $\min_{\tilde{P};\lambda }$ is with respect to the measure $\tilde{P}$, which is associated with the variable $\tilde{\xi }$, and the expectation $E_{P}$ is with respect to P and has an associated variable ξ (that is, the variable ξ in *(27)* is associated with P and the variable $\tilde{\xi }$ with $\tilde{P}$).*

**Remark** **4** (Standard quantization)**.** *The result from *(27)* extends*

(28) $$\begin{array}{cc} \underset{\pi :\begin{array}{c} \pi_{1}=P\;and\\ \mathrm{supp}\;\pi_{2}=\mathrm{supp}\;\tilde{P} \end{array}}{\inf}\; & =\int_{X}\min_{\xi \in \mathrm{supp}\;\tilde{P}}d{\left(\xi ,\tilde{\xi }\right)}^{r}\;P\left(d\xi \right) \end{array}$$

(29) $$\begin{array}{cc} \; & =E_{P}\left(\min_{\tilde{\xi }\in \mathrm{supp}\;\tilde{P}}d{\left(\xi ,\tilde{\xi }\right)}^{r}\right), \end{array}$$

*which is the formula without regularization and with restriction to the marginals P and $\tilde{P}$ (i.e., λ=0, cf. Pflug and Pichler [29]). Note that the preceding display thereby explicitly involves the support $\mathrm{supp}\;\tilde{P}$, while *(26)* only involves the expectation (via the smooth minimum) with respect to the measure $\tilde{P}$. In other words, *(27)* quantifies the quality of entropy-regularized quantization, while *(29)* quantifies standard quantization.*

**Proof of Proposition** **1.** It follows from the definition of the Kullback–Leibler divergence in (4) that it is enough to consider measures π which are absolutely continuous with respect to the product measure $\pi \ll P\times \tilde{P}$; otherwise, the objective is not finite. Hence, there is a Radon–Nikodým density $\tilde{Z}$ such that, with Fubini’s theorem,

$$\pi \left(A\times B\right)=\int_{A}\int_{B}\tilde{Z}\left(\xi ,\eta \right)\;\tilde{P}\left(d\eta \right)P\left(d\xi \right).$$

In order for the marginal constraint π(A×X)=P(A) to be satisfied (cf. (2)), we have

$$\int_{A}\int_{X}\tilde{Z}\left(\xi ,\eta \right)\tilde{P}\left(d\eta \right)\;P\left(d\xi \right)=\pi \left(A\times X\right)=P\left(A\right)=\int_{A}1\;P\left(d\xi \right)$$

for every measurable set *A*. It follows that

$$\int_{X}\tilde{Z}\left(\xi ,\eta \right)\;\tilde{P}\left(d\eta \right)=1\;P\left(d\xi \right)\;\mathrm{almost}\;\mathrm{everywhere}.$$

We can conclude that every density of the form

(30) $$\tilde{Z}\left(\xi ,\eta \right)=\frac{Z(\xi ,\eta )}{\int_{X}Z\left(\xi ,\eta^{\prime }\right)\;\tilde{P}\left(d\eta^{\prime }\right)}$$

satisfies constraints in (2), irrespective of *Z*, and conversely that, via $\tilde{Z}$ in (30), every *Z* defines a bivariate measure π satisfying the constraints in (2). We set Φ(ξ,η):=logZ(ξ,η) (with the convention that log0=−∞ and exp(−∞)=0, resp.) and consider

$$\tilde{Z}\left(\xi ,\eta \right)=\frac{e^{\Phi (\xi ,\eta )}}{\int_{X}e^{\Phi (\xi ,\eta^{\prime })}\;\tilde{P}\left(d\eta^{\prime }\right)}.$$

With these, the divergence is

$$\begin{array}{cc} D(\pi ∥P & \times \tilde{P})=\\ \; & =\int_{X}\int_{X}\frac{e^{\Phi (\xi ,\eta )}}{\int_{X}e^{\Phi (\xi ,\eta^{\prime })}\;\tilde{P}\left(d\eta^{\prime }\right)}\log\frac{e^{\Phi (\xi ,\eta )}\tilde{P}\left(d\eta \right)P\left(d\xi \right)}{\int_{X}e^{\Phi (\xi ,\eta^{\prime })}\;\tilde{P}\left(d\eta^{\prime }\right)P\left(d\xi \right)\tilde{P}\left(d\eta \right)}\tilde{P}\left(d\eta \right)P\left(d\xi \right)\\ \; & =\int_{X}\int_{X}\frac{e^{\Phi (\xi ,\eta )}}{\int_{X}e^{\Phi (\xi ,\eta^{\prime })}\;\tilde{P}\left(d\eta^{\prime }\right)}\log\frac{e^{\Phi (\xi ,\eta )}}{\int_{X}e^{\Phi (\xi ,\eta^{\prime })}\;\tilde{P}\left(d\eta^{\prime }\right)}\tilde{P}\left(d\eta \right)P\left(d\xi \right)\\ \; & =\int_{X}\int_{X}\frac{e^{\Phi (\xi ,\eta )}}{\int_{X}e^{\Phi (\xi ,\eta^{\prime })}\;\tilde{P}\left(d\eta^{\prime }\right)}\Phi \left(\xi ,\eta \right)\;\tilde{P}\left(d\eta \right)P\left(d\xi \right)\\ \; & \;-\int_{X}\frac{e^{\Phi (\xi ,\eta )}}{\int_{X}e^{\Phi (\xi ,\eta^{\prime })}\;\tilde{P}\left(d\eta^{\prime }\right)}\log\int_{X}e^{\Phi (\xi ,\eta^{\prime })}\;\tilde{P}\left(d\eta^{\prime }\right)\tilde{P}\left(d\eta \right)P\left(d\xi \right)\\ \; & =\int_{X}\int_{X}\frac{e^{\Phi (\xi ,\eta )}}{\int_{X}e^{\Phi (\xi ,\eta^{\prime })}\;\tilde{P}\left(d\eta^{\prime }\right)}\Phi \left(\xi ,\eta \right)-\log\int_{X}e^{\Phi (\xi ,\eta^{\prime })}\;\tilde{P}\left(d\eta^{\prime }\right)P\left(d\xi \right). \end{array}$$

For the other term in Objective (24), we have

$$E_{\pi }d^{r}=\int_{X}\int_{X}\frac{e^{\Phi (\xi ,\eta )}}{\int_{X}e^{\Phi (\xi ,\eta^{\prime })}\;\tilde{P}\left(d\eta^{\prime }\right)}d{\left(\xi ,\eta \right)}^{r}\;\tilde{P}\left(d\eta \right)P\left(d\xi \right).$$

Combining the last expressions obtained, the objective in (26) is

(31) $$\begin{array}{cc} E_{\pi }d^{r}+\lambda \;D\left(\pi ∥P\times \tilde{P}\right) & =\int_{X}\int_{X}\frac{e^{\Phi (\xi ,\eta )}}{\int_{X}e^{\Phi (\xi ,\eta^{\prime })}\;\tilde{P}\left(d\eta^{\prime }\right)}\left(d{\left(\xi ,\eta \right)}^{r}+\lambda \;\Phi \left(\xi ,\eta \right)\right)\;\tilde{P}\left(d\eta \right)P\left(d\xi \right) \end{array}$$

(32) $$\begin{array}{cc} \; & \;-\lambda \int_{X}\log\int_{X}e^{\Phi (\xi ,\eta^{\prime })}\;\tilde{P}\left(d\eta^{\prime }\right)P\left(d\xi \right). \end{array}$$

For fixed ξ (ξ is suppressed in the following two displays to abbreviate the notation), consider the function

$$f\left(\Phi \right):=\int_{X}\frac{e^{\Phi (\eta )}}{\int_{X}e^{\Phi (\eta^{\prime })}\;\tilde{P}\left(d\eta^{\prime }\right)}\left(d{\left(\eta \right)}^{r}+\lambda \;\Phi \left(\eta \right)\right)\;\tilde{P}\left(d\eta \right)-\lambda \log\int_{X}e^{\Phi (\eta^{\prime })}\;\tilde{P}\left(d\eta^{\prime }\right).$$

The directional derivative in direction *h* of this function is

(33) $$\begin{array}{c} \lim_{t\to 0}\frac{1}{t}\left(f(\Phi +t\;h)-f(\Phi )\right) \end{array}$$

(34) $$\begin{array}{cc} \; & =\int_{X}\frac{e^{\Phi (\eta )}}{\int_{X}e^{\Phi (\eta^{\prime })}\;\tilde{P}\left(d\eta^{\prime }\right)}\left(d{\left(\eta \right)}^{r}+\lambda \;\Phi \left(\eta \right)-\lambda \right)h\left(\eta \right)\;\tilde{P}\left(d\eta \right) \end{array}$$

(35) $$\begin{array}{cc} \; & \;-\int_{X}\frac{e^{\Phi (\eta )}\int_{X}e^{\Phi (\eta^{\prime })}h\left(\eta^{\prime }\right)\;\tilde{P}\left(d\eta^{\prime }\right)}{{\left(\int_{X}e^{\Phi (\eta^{\prime })}\;\tilde{P}\left(d\eta^{\prime }\right)\right)}^{2}}\left(d{\left(\eta \right)}^{r}+\lambda \;\Phi \left(\eta \right)\right)\;\tilde{P}\left(d\eta \right) \end{array}$$

(36) $$\begin{array}{cc} \; & \;+\lambda \int_{X}\frac{e^{\Phi (\eta )}h\left(\eta \right)}{\int_{X}e^{\Phi (\eta^{\prime })}\;\tilde{P}\left(d\eta^{\prime }\right)}\tilde{P}\left(d\eta \right) \end{array}$$

(37) $$\begin{array}{cc} \; & =\int_{X}\frac{e^{\Phi (\eta )}}{\int_{X}e^{\Phi (\eta^{\prime })}\;\tilde{P}\left(d\eta^{\prime }\right)}\left(d{\left(\eta \right)}^{r}+\lambda \;\Phi \left(\eta \right)\right)h\left(\eta \right)\;\tilde{P}\left(d\eta \right) \end{array}$$

(38) $$\begin{array}{cc} \; & \;-\int_{X}\frac{e^{\Phi (\eta )}\int_{X}e^{\Phi (\eta^{\prime })}h\left(\eta^{\prime }\right)\;\tilde{P}\left(d\eta^{\prime }\right)}{{\left(\int_{X}e^{\Phi (\eta^{\prime })}\;\tilde{P}\left(d\eta^{\prime }\right)\right)}^{2}}\left(d{\left(\eta \right)}^{r}+\lambda \;\Phi \left(\eta \right)\right)\;\tilde{P}\left(d\eta \right). \end{array}$$

Per (37) and (38), the derivative vanishes for every function *h* if $d{\left(\eta \right)}^{r}+\lambda \;\Phi \left(\eta \right)=0$. As ξ is arbitrary, the general minimum is attained for $\Phi \left(\xi ,\eta \right)=-d{\left(\xi ,\eta \right)}^{r}/\lambda$. With this, the first expression in (31) vanishes, and we can conclude that

$$\begin{array}{cc} \underset{\pi }{\inf}E_{\pi }d^{r}+\lambda \;D\left(\pi ∥P\times \tilde{P}\right)\; & =-\lambda \int_{X}\log\int_{X}e^{-d{\left(\xi ,\eta \right)}^{r}/\lambda }\;\tilde{P}\left(d\eta \right)P\left(d\xi \right)\\ \; & =E_{P}\left(\min_{\tilde{P};\lambda }d{\left(\xi ,\tilde{\xi }\right)}^{r}\right). \end{array}$$

Finally, notice that the variable $Z\left(\xi ,\eta \right)=e^{\Phi (\xi ,\eta )}$ is completely arbitrary for the problem in (26) involving the Wasserstein distance and the Kullback–Leibler divergence. As outlined above, for every measure π with finite divergence $D(\pi ∥P\times \tilde{P})$, there is a density *Z*, as considered above. From this, the assertion in Proposition 1 follows.    □

**Remark** **5.** *The preceding proposition considers probability measures π with marginal $\pi_{1}=P$. Its first marginal distribution (trivially) is absolutely continuous with respect to P, $\pi_{1}\ll P$, as $\pi_{1}=P$.*

*The second marginal $\pi_{2}$, however, is not specified. In order for π to be feasible in *(26)*, its Kullback–Leibler divergence with respect to $P\times \tilde{P}$ must be finite. Hence, there is a (non-negative) Radon–Nikodým density Z such that*

$$\pi_{2}\left(B\right)=\pi \left(X\times B\right)=\underset{X\times B}{\;\int \int \;}Z\left(\xi ,\eta \right)P\left(d\xi \right)\tilde{P}\left(d\eta \right).$$

*It follows from Fubini’s theorem that*

$$\pi_{2}\left(B\right)=\int_{B}\int_{X}Z\left(\xi ,\eta \right)\;P\left(d\xi \right)\tilde{P}\left(d\eta \right)=\int_{B}Z\left(\eta \right)\;\tilde{P}\left(d\eta \right),$$

*where $Z\left(\eta \right):=\int_{X}Z\left(\xi ,\eta \right)\;P\left(d\xi \right)$. Thus, the second marginal is absolutely continuous with respect to $\tilde{P}$, $\pi_{2}\ll \tilde{P}$.*

Proposition 1 characterizes the *objective* of the quantization problem. In addition, its proof implicitly reveals the marginal of the best approximation. The following lemma explicitly spells out the density of the marginal of the optimal measure with respect to $\tilde{P}$.

**Lemma** **2** (Characterization of the best approximating measure)**.** *The best approximating marginal probability measure minimizing *(26)* has a density*

$$Z\left(\tilde{\xi }\right)=E_{P}\sigma_{\lambda }\left(d{\left(\xi ,\tilde{\xi }\right)}^{r}\right)=\int_{X}\sigma_{\lambda }\left(d{\left(\xi ,\tilde{\xi }\right)}^{r}\right)\;P\left(d\xi \right),$$

*where $\sigma_{\lambda }\left(\cdot \right)$ is the softmin function (cf. Definition 4).*

**Proof.**  Recall from the proof of Proposition 1 that we have the density

$$\tilde{Z}\left(\xi ,\tilde{\xi }\right)=\frac{e^{-d{\left(\xi ,\tilde{\xi }\right)}^{r}/\lambda }}{E_{\tilde{P}}e^{-d{\left(\xi ,\tilde{\xi }\right)}^{r}/\lambda }}$$

of the optimal measure π relative to $P\times \tilde{P}$. From this, we can derive

$$\pi_{2}\left(B\right)=\pi \left(X\times B\right)=\int_{B}\int_{X}\frac{e^{-d{\left(\xi ,\tilde{\xi }\right)}^{r}/\lambda }}{E_{\tilde{P}}e^{-d{\left(\xi ,\tilde{\xi }\right)}^{r}/\lambda }}\;P\left(d\xi \right)\tilde{P}\left(d\tilde{\xi }\right)$$

such that

$$Z\left(\tilde{\xi }\right)=\int_{X}\frac{e^{-d{\left(\xi ,\tilde{\xi }\right)}^{r}/\lambda }}{E_{\tilde{P}}e^{-d{\left(\xi ,\tilde{\xi }\right)}^{r}/\lambda }}P\left(d\xi \right)=E_{P}\sigma_{\lambda }\left(d{\left(\xi ,\tilde{\xi }\right)}^{r}\right)$$

is the density with respect to $\tilde{P}$, that is, $d\pi_{2}=Z\;d\tilde{P}$ (i.e., $\pi_{2}\left(d\tilde{\xi }\right)=Z\left(\tilde{\xi }\right)\;\tilde{P}\left(d\tilde{\xi }\right)$).    □

### 3.2. Approximation with Flexible Marginal Measure

The following proposition reveals that the best approximation of a bivariate measure in terms of a product of independent measures is provided by the product of its marginals. With this, it follows that the objectives in (25) and (26) coincide for $\tilde{P}=\pi_{2}$.

**Proposition** **2.** *Let P be a measure and let π be a bivariate measure with marginal $\pi_{1}=P$ and $\pi_{2}$. Then, it holds that*

(39) $$D\left(\pi ∥\;P\times \pi_{2}\right)\leq D\left(\pi ∥\;P\times \tilde{P}\right),$$

*where $\tilde{P}$ is an arbitrary measure.*

**Proof.**  Define the Radon–Nikodým density $Z\left(\eta \right):=\frac{\pi_{2}\left(d\eta \right)}{\tilde{P}\left(d\eta \right)}$ and observe that the extension Z(ξ,η):=Z(η) to X×X is the density $Z=\frac{dP\times \pi_{2}}{dP\times \tilde{P}}$. It follows with (4) that

(40) $$\begin{array}{cc} 0\leq D(\pi_{2}∥\;\tilde{P}) & =E_{\pi_{2}}\log\frac{d\pi_{2}}{d\tilde{P}} \end{array}$$

(41) $$\begin{array}{cc} \; & =E_{\pi }\log\frac{d\;P\times \pi_{2}}{d\;P\times \tilde{P}} \end{array}$$

(42) $$\begin{array}{cc} \; & =E_{\pi }\left(\log\frac{d\;\pi }{d\;P\times \tilde{P}}-\log\frac{d\;\pi }{d\;P\times \pi_{2}}\right) \end{array}$$

(43) $$\begin{array}{cc} \; & =D\left(\pi ∥\;P\times \tilde{P}\right)-D\left(\pi ∥\;P\times \pi_{2}\right), \end{array}$$

which is the assertion. In case the measures are not absolutely continuous, the assertion in (40) is trivial.    □

Suppose now that π is a solution of the master problem (26) with some $\tilde{P}$. It follows from the preceding proposition that the objective (26) improves when replacing the initial $\tilde{P}$ with the marginal of the optimal solution, that is, $\tilde{P}=\pi_{2}$.

### 3.3. The Relation of Soft Quantization and Entropy

The soft quantization problem (26) involves the Kullback–Leibler divergence and *not* the entropy. The major advantage of the formulation presented above is that it works for discrete, continuous, or mixed measures, while entropy usually needs to be defined separately for discrete and continuous measures.

For a discrete measure with P(x):=P({x}) and $\tilde{P}\left(y\right):=\tilde{P}\left(\left\{y\right\}\right)$, the Kullback–Leibler divergence (4) is

(44) $$\begin{array}{cc} D(\tilde{P}∥\;P) & =H\left(\tilde{P},P\right)-H\left(\tilde{P}\right) \end{array}$$

(45) $$\begin{array}{cc} \; & =\sum_{x\in X}\tilde{P}\left(x\right)\log\frac{\tilde{P}\left(x\right)}{P(x)}, \end{array}$$

where

$$H\left(\tilde{P},P\right):=-\sum_{x\in X}\tilde{P}\left(x\right)\cdot \log P\left(x\right)$$

is the *cross-entropy* of the measures $\tilde{P}$ and *P*, while 

(46) $$H\left(\tilde{P}\right):=H\left(\tilde{P},\tilde{P}\right)=-\sum_{x\in X}\tilde{P}\left(x\right)\log\tilde{P}\left(x\right)$$

is the *entropy* of $\tilde{P}$.

For a measure π with marginals *P* and $\tilde{P}$, the cross-entropy is

(47) $$\begin{array}{cc} H(\pi ,P\times \tilde{P}) & =-\sum_{x,y}\pi \left(x,y\right)\log\left(P\left(x\right)\cdot \tilde{P}\left(y\right)\right) \end{array}$$

(48) $$\begin{array}{cc} \; & =-\sum_{x,y}\pi \left(x,y\right)\log P\left(x\right)-\sum_{x,y}\pi \left(x,y\right)\log\tilde{P}\left(y\right) \end{array}$$

(49) $$\begin{array}{cc} \; & =-\sum_{x}P\left(x\right)\log P\left(x\right)-\sum_{y}\tilde{P}\left(y\right)\log\tilde{P}\left(y\right), \end{array}$$

where we have used the marginals from (2). Note that (49) does not depend on π; hence, $H(\pi ,P\times \tilde{P})$ does not depend on π.

With (44), the quantization problem (26) can be rewritten equivalently as

(50) $$\min_{\pi :\pi_{2}\in P}\underset{X\times X}{\;\int \int \;}d^{r}\;d\pi -\lambda \cdot H\left(\pi \right)$$

by involving the entropy only. For this reason, we call the master problem in (26) the *entropy-regularized problem*.

## 4. Soft Tessellation

The quantization problem (25) consists of finding a good (in the best case, the optimal) approximation of a general probability measure *P* on X using a simple and discrete measure ${\tilde{P}}_{m}=\sum_{j=1}^{m}{\tilde{p}}_{j}\;\delta_{y_{j}}$. Thus, the problem consists of finding good weights ${\tilde{p}}_{1},\ldots ,{\tilde{p}}_{m}$ as well as good locations $y_{1},\ldots ,y_{m}$. Quantization employs the Wasserstein distance to measure the quality of the approximation; instead, soft quantization involves the regularized Wasserstein distance, as in (26):

$$\underset{{\tilde{P}}_{m}\in P_{m}\left(X\right)}{\inf}\;\underset{\pi :\begin{array}{c} \pi_{1}=P,\\ \pi_{2}={\tilde{P}}_{m} \end{array}}{\inf}E_{\pi }d^{r}+\lambda \cdot D\left(\pi ∥\;P\times {\tilde{P}}_{m}\right),$$

where the measures on X supported by not more than *m* points (cf. (23)) are as follows:

$$P_{m}\left(X\right)=\left\{{\tilde{P}}_{m}\in P\left(X\right):{\tilde{P}}_{m}=\sum_{j=1}^{m}{\tilde{p}}_{j}\;\delta_{y_{j}}\right\}.$$

We separate the problems of finding the best weights and locations. The following Section 4.1 addresses the problem of finding the optimal weights $\tilde{p}$; the subsequent Section 4.2 then addresses the problem of finding the optimal locations $y_{1},\ldots ,y_{m}$. As well, we elaborate the numerical advantages of *soft* quantization below.

### 4.1. Optimal Weights

Proposition 1 above is formulated for the general probability measures *P* and $\tilde{P}$. The desired measure in quantization is a simple and discrete measure. To this end, recall that, per Remark 5, measures which are feasible for (26) have marginals $\pi_{2}$ with $\pi_{2}\ll \tilde{P}$. It follows that the support of the marginal is smaller than the support of $\tilde{P}$, that is,

$$\mathrm{supp}\;\pi_{2}\subset \mathrm{supp}\;\tilde{P}.$$

For a simple measure $\tilde{P}=\sum_{j=1}^{m}{\tilde{p}}_{j}\;\delta_{y_{j}}$ with ${\tilde{p}}_{j}>0$, it follows in particular that $\mathrm{supp}\;\pi_{2}\subset \left\{y_{1},\ldots ,y_{m}\right\}$. In this subsection, we consider the measure $\tilde{P}$ and the support $\left\{y_{1},\ldots ,y_{m}\right\}$ to be fixed.

To unfold the result of Proposition 1 for discrete measures, recall the smooth minimum and the softmin function for the discrete (empirical or uniform) measure $\tilde{P}=\sum_{j=1}^{m}{\tilde{p}}_{j}\;\delta_{y_{j}}$. For this measure, the smooth minimum (6) explicitly is

$$\min_{\lambda ;\tilde{P}}\left(y_{1},\ldots ,y_{m}\right)=-\lambda \log\left({\tilde{p}}_{1}\;e^{-y_{1}/\lambda }+\ldots +{\tilde{p}}_{m}\;e^{-y_{m}/\lambda }\right).$$

This function is occasionally referred to as the LogSumExp function. The softmin function (or Gibbs density (12)) is

$$\sigma_{\lambda }\left(y_{1},\ldots ,y_{m}\right)={\left(\frac{e^{-y_{j}/\lambda }}{{\tilde{p}}_{1}\;e^{-y_{1}/\lambda }+\ldots +{\tilde{p}}_{m}\;e^{-y_{m}/\lambda )}}\right)}_{j=1}^{m}.$$

It follows from Lemma 2 that the best approximating measure is $Q=\sum_{j=1}^{m}q_{j}\;{\tilde{p}}_{j}\;\delta_{y_{j}}$, where the vector *q* of the optimal weights relative to $\tilde{P}$ is provided explicitly by

(51) $$q=\int_{X}\sigma_{\lambda }\left(d{\left(\xi ,y_{1}\right)}^{r},\ldots ,d{\left(\xi ,y_{m}\right)}^{r}\right)\;P\left(d\xi \right)=E_{P}\sigma_{\lambda }\left(d{\left(\xi ,y_{1}\right)}^{r},\ldots ,d{\left(\xi ,y_{m}\right)}^{r}\right),$$

which involves computing expectations.

**Soft Tessellation**

For λ=0, the softmin function $\sigma_{\lambda }$ is

$${\tilde{p}}_{j}\cdot \sigma_{\lambda =0}{\left(d{\left(\xi ,y_{1}\right)}^{r},\ldots ,d{\left(\xi ,y_{m}\right)}^{r}\right)}_{j}=\left\{\begin{array}{cc} 1 & \mathrm{if}\;d{\left(\xi ,y_{j}\right)}^{r}=\min\left(d{\left(\xi ,y_{1}\right)}^{r},\ldots ,d{\left(\xi ,y_{m}\right)}^{r}\right),\\ 0 & \mathrm{else}. \end{array}\right.$$

That is, the mapping $j\mapsto {\tilde{p}}_{j}\cdot \sigma_{\lambda }{\left(\ldots \right)}_{j}$ can serve for classification, i.e., tessellation; the point ξ is associated with $y_{j}$ if $\sigma_{\lambda }{\left(\ldots \right)}_{j}\neq 0$, and the corresponding region is known as a Voronoi diagram.

For λ>0, the softmin ${\tilde{p}}_{j}\cdot \sigma_{\lambda }{\left(\ldots \right)}_{j}$ is not a strict indicator, and can instead be interpreted as probability; that is,

$${\tilde{p}}_{j}\cdot \sigma_{\lambda }{\left(d{\left(\xi ,y_{1}\right)}^{r},\ldots ,d{\left(\xi ,y_{m}\right)}^{r}\right)}_{j}$$

is the probability of allocating ξ∈X to the quantizer $y_{j}$.

**Remark** **6** (K-means and quantization)**.** *K-means clustering is a widely used unsupervised machine learning algorithm that groups datapoints into clusters based on their similarity. Voronoi tessellation, on the other hand, is a geometrical concept used to partition a space into regions, each of which are associated with a specific point or seed. Notably, this is an unavoidable concept in the investigation of optimal quantizers. In the K-means context, Voronoi tessellation helps to define cluster boundaries. Each cluster’s boundary is constructed as the region within which the datapoints are closer to its cluster center than to any other (cf. Graf and Luschgy [4] Chapter I).*

### 4.2. Optimal Locations

As a result of Proposition 1, the objective in (27) is an expectation. To identify the optimal support points $y_{1},\ldots ,y_{m}$, it is essential to first minimize

(52) $$\min_{\tilde{P}=\sum_{j=1}^{m}{\tilde{p}}_{j}\delta_{y_{j}}}E_{\xi ∼P}\left(\min_{\lambda ;y∼\tilde{P}}d{\left(\xi ,y\right)}^{r}\right).$$

This is a stochastic, nonlinear, and non-convex optimization problem:

(53) $$f\left(y_{1},\ldots ,y_{m}\right):=Ef\left(y_{1},\ldots ,y_{m};\xi \right)=E\left(\underset{j=1,\ldots ,m}{\min_{\lambda ;\tilde{P}}}d{\left(\xi ,y_{i}\right)}^{r}\right),$$

where the function $f\left(y_{1},\ldots ,y_{m};\xi \right):=\min_{\lambda ;\tilde{P}}\left\{d{\left(\xi ,y_{i}\right)}^{r}:j=1,\ldots ,m\right\}$ is nonlinear and non-convex. The optimal quantization problem constitutes an unconstrained, stochastic, non-convex, and nonlinear optimization problem. According to the chain rule and gradients in Section 2.3, the gradient of the objective is constructed from the components

(54) $$\frac{\partial }{\partial y_{j}}f\left(y_{1},\ldots ,y_{m}\right)=\frac{{\tilde{p}}_{j}\cdot \exp\left(-d{\left(\xi ,y_{j}\right)}^{r}/\lambda \right)}{\sum_{j^{\prime }=1}^{m}{\tilde{p}}_{j^{\prime }}\cdot \exp\left(-d{\left(\xi ,y_{j^{\prime }}\right)}^{r}/\lambda \right)}\cdot {\left.\nabla_{y}\;d{\left(\xi ,y\right)}^{r}\right|}_{y=y_{j}},$$

that is,

(55) $$\nabla f=\tilde{p}\cdot \sigma_{\lambda }\left(d{\left(\xi ,y_{1}\right)}^{r},\ldots ,d{\left(\xi ,y_{m}\right)}^{r}\right)\cdot r\;d{\left(\xi ,y\right)}^{r-1}\cdot \nabla_{y}\;d\left(\xi ,y\right),$$

where ‘·’ denotes the Hadamard (element-wise) product and $\tilde{p}$, $d{\left(\xi ,y\right)}^{r-1}$ are the vectors with entries ${\tilde{p}}_{j}$, $d{\left(\xi ,y_{j}\right)}^{r-1}$, j=1,…,m. In other words, the gradient of the LogSumExp function (53) is the softmin function, which Section 2.3 explicitly illustrates.

Algorithm 1 is a stochastic gradient algorithm used to minimize (51), which collects the elements of the optimal weights and the optimal locations provided here and in the preceding section.
**Algorithm 1:** Stochastic gradient algorithm to find the optimal quantizers and optimal masses
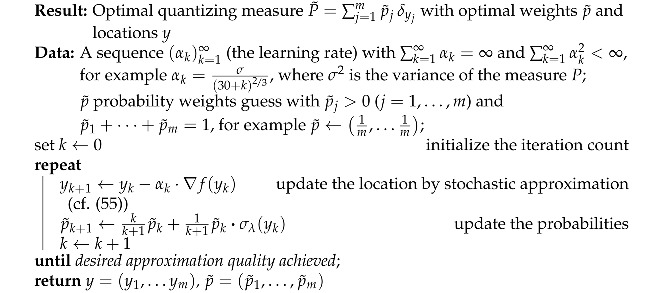

**Example** **1.** *To provide an example of the gradient of the distance function in *(54)* (*(55)*, resp.), the derivative of the weighted norm*

$$d\left(\xi ,y\right)={∥y-\xi ∥}_{p}:={\left(\sum_{\ell =1}^{d}w_{\ell }\cdot {\left|y_{\ell }-\xi_{\ell }\right|}^{p}\right)}^{1/p}$$

*is*

$$\frac{\partial }{\partial y_{j}}{∥y-\xi ∥}_{p}^{r}=r\;w_{j}{\;∥\xi -y∥}_{p}^{\frac{r-p}{p}}\cdot {\left|y_{j}-\xi_{j}\right|}^{p-1}\cdot \mathrm{sign}\left(y_{j}-\xi_{j}\right).$$

### 4.3. Quantization with Large Regularization Parameters

The entropy in (46) is minimal for the Dirac measure $P=\delta_{x}$ (where *x* is any point in X); in this case, $H(\delta_{x})=1\cdot \log1=0$, while $H(\tilde{P})>0$ for any other measure. For larger values of λ, the objective in (50), and as such the objective of the master problem (23), will supposedly prefer a measure with fewer points. This is indeed the case, as stated by Theorem 1 above. We provide its proof below after formally defining the center of the measure.

**Definition** **5** (Center of the measure)**.** *Let P be a probability measure on X and let d be a distance on X. The point a∈X is a* center of the measure *P* with respect to the distance *d if*

$$a\in \underset{x\in X}{arg min}Ed{\left(x,\xi \right)}^{r},$$

*provided that $Ed{\left(x_{0},\xi \right)}^{r}<\infty$ for some (i.e., any) $x_{0}\in X$ and r≥1.*

In what follows, we demonstrate that the regularized quantization problem (50) links the optimal quantization problem and the center of the measure.

**Proof of Theorem** **1.** According to Proposition 1, Problems (50) and (26) are equivalent. Now, assume that $y_{i}=y_{j}$ for all *i*, j≤m; then, $d\left(y_{i},\xi \right)=d\left(y_{j},\xi \right)$ for ξ∈Ξ, and it follows that

$$\min_{\lambda }\left(d{\left(y_{1},\xi \right)}^{r},\ldots ,d{\left(y_{m},\xi \right)}^{r}\right)=d{\left(y_{i},\xi \right)}^{r},\;i=1,\ldots ,m.$$

Thus, the minimum of the optimization problem is attained at $y_{i}=a$ for each i=1,…,m, where *a* is the center of the measure *P* with respect to the distance *d*. It follows that $y_{1}=\ldots =y_{m}=a$ is a local minimum and a stationary point satisfying the first order conditions

$$\nabla f(y_{1},\ldots ,y_{m})=0$$

for the function *f* provided in (53). Note as well that

$$\sigma_{\lambda }{\left(d{\left(\xi ,y_{1}\right)}^{r},\ldots ,d{\left(\xi ,y_{n}\right)}^{r}\right)}_{i}=\frac{\exp\left(-d{\left(\xi ,y_{i}\right)}^{r}/\lambda \right)}{\sum_{j=1}^{n}{\tilde{p}}_{j}\exp\left(-d{\left(\xi ,y_{j}\right)}^{r}/\lambda \right)}=1,$$

and as such the softmin function does not depend on λ at the stationary point $y_{1}=\ldots =y_{m}=a$.

Recall from (54) that

$$\nabla E\left(\underset{j=1,\ldots ,n}{\min_{\lambda ;\tilde{P}}}d{\left(\xi ,y_{j}\right)}^{r}\right)=E\sigma_{\lambda }\left(d{\left(y_{1},\xi \right)}^{r},\ldots ,d{\left(y_{n},\xi \right)}^{r}\right)\cdot \nabla d{\left(\xi ,y_{i}\right)}^{r}.$$

According to the product rule, the Hessian matrix is

(56) $$\nabla^{2}E\left(\underset{j=1,\ldots ,n}{\min_{\lambda ;\tilde{P}}}d{\left(\xi ,y_{j}\right)}^{r}\right)=E\left(\begin{array}{c} \begin{array}{c} \nabla \sigma_{\lambda }\left(d{\left(y_{1},\xi \right)}^{r},\ldots ,d{\left(y_{n},\xi \right)}^{r}\right)\cdot {\left(\nabla d{\left(\xi ,y_{i}\right)}^{r}\right)}^{2}\\ \;+\sigma_{\lambda }\left(d{\left(y_{1},\xi \right)}^{r},\ldots ,d{\left(y_{n},\xi \right)}^{r}\right)\cdot \nabla^{2}d{\left(\xi ,y_{i}\right)}^{r} \end{array} \end{array}\right).$$

Note that the second expression is positive definite, as the Hessian $\nabla^{2}d{\left(\xi ,y_{i}\right)}^{r}$ of the convex function is positive definite and $\nabla {\min_{\lambda ;\tilde{P}}}_{j=1,\ldots ,n}\left(x_{1},\ldots ,x_{n}\right)=\sigma_{\lambda }\left(x_{1},\ldots ,x_{n}\right)\geq 0$. Further, the Hessian of the smooth minimum (see Appendix A) is

$$\nabla \sigma_{\lambda }=\nabla^{2}\underset{j=1,\ldots ,n}{\min_{\lambda }}=-\frac{1}{\lambda }\;\Sigma ,$$

where the matrix Σ is

$$\Sigma :=\mathrm{diag}\left(\sigma_{1},\ldots ,\sigma_{n}\right)-\sigma \sigma^{⊤}.$$

This matrix Σ is positive definite (as $\sum_{i=1}^{n}\sigma_{i}=1$) and 0≤Σ≤1 in Loewner order; indeed, Σ is the covariance matrix of the multinomial distribution. It follows that the first term in (56) is O(1), while the second is $O\left(\frac{1}{\lambda }\right)$, such that (56) is positive definite for sufficiently small λ. Thus, the extremal point $y_{i}=a$ is a minimum for all λ. In particular, there exists $\lambda_{0}>0$ such that (56) is positive definite for every $\lambda >\lambda_{0}$, hence, the result. □

## 5. Numerical Illustration

This section presents numerical findings for the approaches and methods discussed earlier. The Julia implementations for these methods are available online (cf. https://github.com/rajmadan96/SoftQuantization.git, accessed on 8 September 2023).

In the following experiments, we approximate the measure *P* with a finite discrete measure $\tilde{P}$ using the stochastic gradient algorithm presented in Algorithm 1.

### 5.1. One Dimension

First, we perform the analysis in one dimension. In this experiment, our problem of interest is to find entropy-regularized optimal quantizers for

P∼N(0,1)andP∼Exp(1)

(i.e., the normal and exponential distributions with standard parameters). To enhance the peculiarity, we consider only m=8 quantizers.

**Figure 1:**
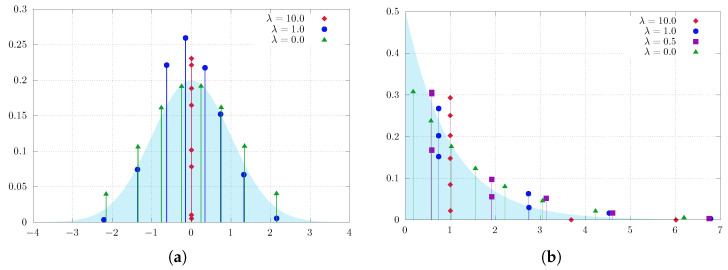
Soft quantization of measures on R with a varying regularization parameter λ with eight quantization points. (**a**) Normal distribution: for λ=10, the best approximation resides at the center of the measure; for λ=1, the approximation is reduced to only six points, as two of the remaining points have probability 0; for λ=0, we obtain the standard quantization. (**b**) Exponential distribution: the measures concentrate on one (λ large), three (λ=1), five (λ=0.5), and eight quantization points.

Figure 1 illustrates the results of soft quantization of the standard normal distribution and exponential distribution. It is apparent that when λ is increased beyond a certain threshold (cf. Theorem 1), the quantizers converge towards the center of the measure (i.e., the mean), while for smaller values of λ the quantizers are able to identify the actual optimal locations with greater accuracy. Furthermore, we emphasize that our proposed method is capable of identifying the mean location regardless of the shape of the distribution, which this experiment empirically substantiates.

For better understanding of the dissemination of the weights (probabilities) and their respective positions, the following examination involves the calculation of the cumulative distribution function. Additionally, we consider

P∼Γ(2,2) (Gammadistribution)

as a problem of interest, which is a notably distinct scenario in terms of shape compared to the measures examined previously.

**Figure 2:**
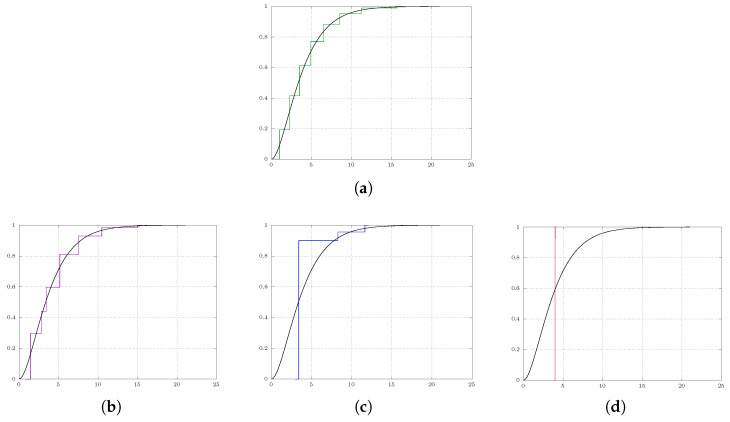
Soft quantization of the Gamma distribution on R with varying regularization parameter λ; the approximating measure is simplifies with increasing λ. (**a**) λ=0: approximate solution to standard quantization problem with eight quantizers. (**b**) λ=1: the eight quantization points collapse to seven quantization points. (**c**) λ=10: the eight quantization points collapse to three quantization points. (**d**) λ=20: the quantization points converge to a single point representing the center of the measure.

Figure 2 provides the results. It is evident that the number of quantizers *m* decreases as λ increases. When λ reaches a specific threshold, such as λ=20 in our case, all quantizers converge towards the center of the measures, represented by the mean (i.e., 4).

### 5.2. Two Dimensions

Next, we demonstrate the behavior of entropy-regularized optimal quantization for a range of λ in two dimensions. In the following experiment, we consider

$$P∼U\left((0,1)\times (0,1)\right)\;(\mathrm{uniform}\;\mathrm{distribution}\;\mathrm{on}\;\mathrm{the}\;\mathrm{square})$$

as a problem of interest. Initially, we perform the experiment with m=4 quantizers.

**Figure 3:**
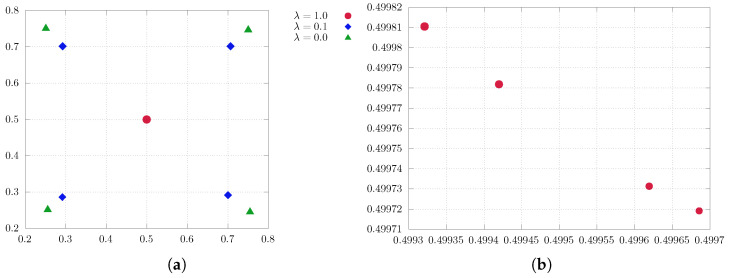
Two-dimensional soft quantization of the uniform distribution on $R^{2}$ with a varying regularization parameter λ with 4 quantizers. (**a**) Uniform distribution in $R^{2}$. (**b**) Enlargement of (**a**): for larger values of λ (here, λ=1), the quantizers align while converging to the center of the measure.

Figure 3 illustrates the findings. Figure 3a reveals a quantization pattern similar to that observed in the one-dimensional experiment. However, in Figure 3b we gain more detailed insight into the behavior of the quantizers at λ=1, where they align diagonally before eventually colliding. Furthermore, the size of the point indicates the respective probability of the quantization point, which is notably uniformly distributed for a varying regularization parameter λ.

**Figure 4:**
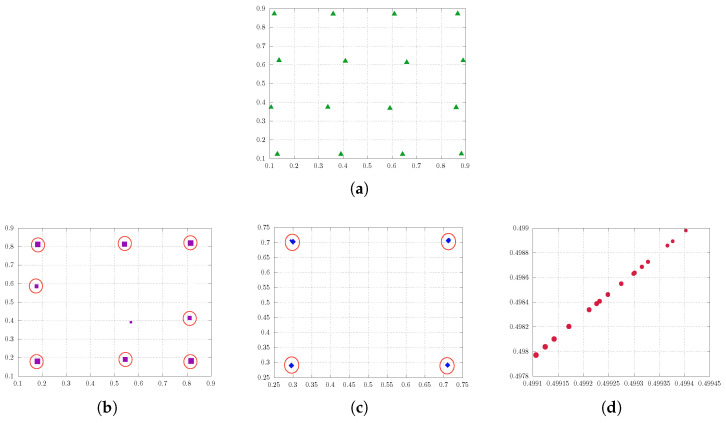
Soft quantization of uniform distribution on $R^{2}$ with varying regularization parameter λ; the approximating measure simplifies with λ increasing. (**a**) λ=0.0: approximate solution to the standard quantization problem with sixteen quantizers. (**b**) λ=0.037: the sixteen quantization points collapse to eight quantization points. (**c**) λ=0.1: the sixteen quantization points collapse to four quantization points. (**d**) λ=1.0: the quantization points converge to a single point, representing the center of the measure, in an aligned way.

Again, we consider a uniform distribution as a problem of interest in the subsequent experiment, this time employing m=16 quantizers for enhanced comprehension. Figure 4 encapsulates the essence of the experiment, offering an extensive visual representation. In contrast to the previous experiment, it can be observed that for regularization values of λ=0.037 and λ=0.1 they assemble at the nearest strong points (in terms of high probability) rather than converging towards the center of the measure (see Figure 4b,c). Subsequently, for larger λ, they move from these strong points towards the center, where they are in a diagonal alignment before colliding (see Figure 4d). More concisely, when λ=0 we achieve the genuine quantization solution (see Figure 4a). As λ increases, the quantizers with lower probabilities converge towards those with nearest higher probabilities. Subsequently, all quantizers converge towards the center of the measure, represented by the mean of respective measure.

Thus far, we have conducted two-dimensional experiments employing various quantizers (m=4 and m=16) with the uniform distribution. These experiments can be categorized under the k-means approach (see Remark 6). Next, we delve into the complexity of a multivariate normal distribution, with the aim of enhancing comprehension. More precisely, our problem of interest is to find a soft quantization for

P∼N(μ,Σ),

where

$$\mu =\left(\begin{array}{c} 0\\ 0 \end{array}\right),\;\Sigma =\left(\begin{array}{cc} 3 & 1\\ 1 & 3 \end{array}\right).$$

**Figure 5:**
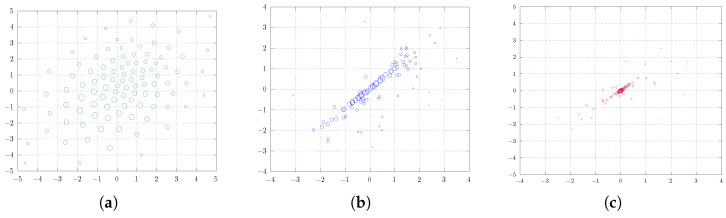
Two-dimensional soft quantization of the normal distribution on $R^{2}$ with varying regularization parameter λ and parameters r=2, p=2, and m=100. (**a**) λ=0.0 , the solution to the standard quantization problem, (**b**) λ=5.0, (**c**) λ=10.0.

In this endeavor, we employ more quantizers, specifically, m=100. Figure 5 captures the core essence of the experiment, delivering a comprehensive and visually illustrative representation. From the experiment, it is evident that the initial diagonal alignment precedes convergence toward the center of the measure as λ increases. Additionally, a noticeable shift can be observed on the part of the points with lower probabilities towards those with higher probabilities. This experiment highlights that the threshold of λ for achieving convergence or diagonal alignment in the center of the measure depends on the number of quantizers employed.

## 6. Summary

In this study, we have enhanced the stability and simplicity of the standard quantization problem by introducing a novel method of quantization using entropy. Propositions 1 and 2 thoroughly elucidate the intricacies of the master problem (25). Our substantiation of the convergence of quantizers to the center of the measure explains the transition from a complex hard optimization problem to a simplified configuration (see Theorem 1). More concisely, this transition underscores the fundamental shift towards a more tractable and straightforward computational framework, marking a significant advancement in terms of the overall approach. Moreover, in Section 5, we provide numerical illustrations of our method that confirm its robustness, stability, and properties, as discussed in our theoretical results. These numerical demonstrations serve as empirical evidence reinforcing the efficacy of our proposed approach.

## Data Availability

Data is available at https://github.com/rajmadan96/SoftQuantization.git, accessed on 8 September 2023.

## Appendix A. Hessian of the Softmin

The empirical measure $\frac{1}{\lambda }\sum_{i=1}^{n}\delta_{x_{i}}$ is a probability measure. From Jensen’s inequality, it follows that $\min_{\lambda }\left(x_{1},\ldots ,x_{n}\right)\leq \frac{1}{n}\sum_{i=1}^{n}x_{i}{\bar{x}}_{n}$. Thus, the smooth minimum involves a cumulant generating function, for which we derive

(A1) $$\begin{array}{cc} \min_{\lambda }(x_{1},\ldots ,x_{n}) & =\sum_{j=1}^{\infty }\frac{{\left(-1\right)}^{j-1}}{\lambda^{j-1}\cdot j!}\kappa_{j} \end{array}$$

(A2) $$\begin{array}{cc} \; & ={\bar{x}}_{n}-\frac{1}{2\lambda }s_{n}^{2}+\frac{1}{6\lambda^{2}}\kappa_{3}+O\left(\lambda^{-3}\right), \end{array}$$

where $\kappa_{j}$ is the *j*-th cumulant with respect to the empirical measure. Specifically,

$$\kappa_{1}={\bar{x}}_{n}=\frac{1}{n}\sum_{i=1}^{n}x_{i},\;\kappa ₂=s_{n}^{2}=\frac{1}{n}\sum_{i=1}^{n}{\left(x_{i}-{\bar{x}}_{n}\right)}^{2},\;\kappa_{3}=\frac{1}{n}\sum_{i=1}^{n}{\left(x_{i}-{\bar{x}}_{n}\right)}^{3},$$

where ${\bar{x}}_{n}$ is the ‘sample mean’ and $s_{n}^{2}$ the ‘sample variance’. The following cumulants (κ₄, etc.) are more involved. The Taylor series expansion $\log\left(1+x\right)=x-\frac{1}{2}x^{2}+O\left(x^{3}\right)$ and

$$\begin{array}{cc} -\lambda \log\frac{1}{n}\sum_{i=1}^{n}e^{-x_{i}/\lambda }\; & ={\bar{x}}_{n}-\lambda \log\frac{1}{n}\sum_{i=1}^{n}e^{-(x_{i}-{\bar{x}}_{n})/\lambda }\\ \; & ={\bar{x}}_{n}-\lambda \log\sum_{i=1}^{n}\frac{1}{n}\left(1-\frac{x_{i}-{\bar{x}}_{n}}{\lambda }+\frac{1}{2}{\left(\frac{x_{i}-x_{n}}{\lambda }\right)}^{2}-\frac{1}{6}\left(\frac{x_{i}-{\bar{x}}_{n}}{\lambda^{3}}\right)+O\left(\frac{1}{\lambda ⁴}\right)\right)\\ \; & ={\bar{x}}_{n}-\lambda \log\left(1+\frac{1}{2\lambda^{2}}s_{n}^{2}-\frac{1}{6\lambda^{3}}\kappa_{3}+O\left(\lambda^{-4}\right)\right)\\ \; & ={\bar{x}}_{n}-\lambda \left(\frac{1}{2\lambda^{2}}s_{n}^{2}-\frac{1}{6\lambda^{3}}\kappa_{3}+O\left(\lambda^{-4}\right)\right)\\ \; & ={\bar{x}}_{n}-\frac{1}{2\lambda }s_{n}^{2}+\frac{1}{6\lambda^{2}}\kappa_{3}+O\left(\lambda^{3}\right). \end{array}$$

Note as well that the softmin function is the gradient of the smooth minimum:

$$\sigma_{\lambda }{\left(x_{1},\ldots ,x_{m}\right)}_{i}=\frac{\partial }{\partial x_{i}}\min_{\lambda }\left(x_{1},\ldots ,x_{n}\right).$$

The softmin function is frequently used in classification in a maximum likelihood framework. It holds that

$$\begin{array}{cc} \frac{\partial^{2}}{\partial x_{i}\partial x_{j}}\min_{\lambda }\left(x_{1},\ldots ,x_{n}\right)\; & =\frac{\partial }{\partial x_{j}}\frac{\exp(-\lambda \;x_{i})}{\sum_{k=1}^{m}\exp\left(-\lambda \;x_{k}\right)}\\ \; & =+\lambda \frac{\exp(-\lambda \;x_{i}-\lambda \;x_{j})}{{\left(\sum_{k=1}^{m}\exp\left(-\lambda \;x_{k}\right)\right)}^{2}}\\ \; & =\lambda \;\sigma_{i}\sigma_{j} \end{array}$$

for i≠j and

$$\begin{array}{cc} \frac{\partial^{2}}{\partial x_{i}^{2}}\min_{\lambda }\left(x_{1},‥,x_{n}\right)\; & =\frac{\partial }{\partial x_{i}}\frac{\exp(-\lambda \;x_{i})}{\sum_{j=1}^{m}\exp\left(-\lambda \;x_{j}\right)}\\ \; & =\frac{-\lambda \exp\left(-\lambda \;x_{i}\right)\left(\sum_{j=1}^{m}\exp\left(-\lambda \;x_{j}\right)\right)+\lambda \exp\left(-\lambda \;x_{i}-\lambda \;x_{i}\right)}{\left(\sum_{j=1}^{m}\exp\left(-\lambda \;x_{j}\right)\right)}\\ \; & =-\lambda \;\sigma_{i}+\lambda \sigma_{i}\sigma_{i}, \end{array}$$

that is,

$$\nabla^{2}\min_{\lambda }\left(x_{1},\ldots ,x_{n}\right)=\lambda \left(\sigma \sigma^{⊤}-\mathrm{diag}\sigma \right)=-\lambda \cdot \left(\left(\begin{array}{ccc} \sigma_{1} & 0 & \ddots \\ 0 & \ddots & 0\\ \ddots & 0 & \sigma_{n} \end{array}\right)-\sigma \cdot \sigma^{⊤}\right).$$

## References

[1] Graf S., Mauldin R.D. (1989). A Classification of Disintegrations of Measures. Contemp. Math..

[2] Luschgy H., Pagès G. (2015). Greedy vector quantization. J. Approx. Theory.

[3] El Nmeir R., Luschgy H., Pagès G. (2022). New approach to greedy vector quantization. Bernoulli.

[4] Graf S., Luschgy H. (2000). Foundations of Quantization for Probability Distributions.

[5] Breuer T., Csiszár I. (2013). Measuring distribution model risk. Math. Financ..

[6] Breuer T., Csiszár I. (2013). Systematic stress tests with entropic plausibility constraints. J. Bank. Financ..

[7] Pichler A., Schlotter R. (2020). Entropy based risk measures. Eur. J. Oper. Res..

[8] Jacob B., Kligys S., Chen B., Zhu M., Tang M., Howard A., Adam H., Kalenichenko D. Quantization and training of neural networks for efficient integer-arithmetic-only inference. Proceedings of the IEEE Conference on Computer Vision and Pattern Recognition (CVPR).

[9] Zhuang B., Liu L., Tan M., Shen C., Reid I. (2020). Training quantized neural networks with a full-precision auxiliary module. Proceedings of the IEEE/CVF Conference on Computer Vision and Pattern Recognition.

[10] Hubara I., Courbariaux M., Soudry D., El-Yaniv R., Bengio Y. (2016). Binarized neural networks. Adv. Neural Inf. Process. Syst..

[11] Polino A., Pascanu R., Alistarh D.-A. Model compression via distillation and quantization. Proceedings of the 6th International Conference on Learning Representations.

[12] Bhattacharya K. (2023). Semi-classical description of electrostatics and quantization of electric charge. Phys. Scr..

[13] Scheunders P. (1996). A genetic Lloyd-Max image quantization algorithm. Pattern Recognit. Lett..

[14] Wei L.Y., Levoy M. (2000). Fast texture synthesis using tree-structured vector quantization. Proceedings of the 27th Annual Conference on Computer Graphics and Interactive Techniques.

[15] Heskes T. (2001). Self-organizing maps, vector quantization, and mixture modeling. IEEE Trans. Neural Netw..

[16] Pagès G., Pham H., Printems J. (2004). Optimal Quantization Methods and Applications to Numerical Problems in Finance. Handbook of Computational and Numerical Methods in Finance.

[17] Cuturi M. Sinkhorn distances: Lightspeed computation of optimal transport. Proceedings of the 26th International Conference on Neural Information Processing Systems.

[18] Ramdas A., García Trillos N., Cuturi M. (2017). On Wasserstein two-sample testing and related families of nonparametric tests. Entropy.

[19] Neumayer S., Steidl G. (2021). From optimal transport to discrepancy. Handbook of Mathematical Models and Algorithms in Computer Vision and Imaging: Mathematical Imaging and Vision.

[20] Altschuler J., Bach F., Rudi A., Niles-Weed J., Wallach H., Larochelle H., Beygelzimer A., d’ Alché-Buc F., Fox E., Garnett R. (2019). Massively scalable Sinkhorn distances via the Nyström method. Advances in Neural Information Processing Systems.

[21] Lakshmanan R., Pichler A., Potts D. (2023). Nonequispaced Fast Fourier Transform Boost for the Sinkhorn Algorithm. Etna—Electron. Trans. Numer. Anal..

[22] Ba F.A., Quellmalz M. (2022). Accelerating the Sinkhorn algorithm for sparse multi-marginal optimal transport via fast Fourier transforms. Algorithms.

[23] Lakshmanan R., Pichler A. (2023). Fast approximation of unbalanced optimal transport and maximum mean discrepancies. arXiv.

[24] Monge G. (1781). Mémoire sue la théorie des déblais et de remblais. Histoire de l’Académie Royale des Sciences de Paris, Avec les Mémoires de Mathématique et de Physique Pour la Même Année.

[25] Kantorovich L. (2006). On the translocation of masses. J. Math. Sci..

[26] Villani C. (2003). Topics in Optimal Transportation.

[27] Rachev S.T., Rüschendorf L. (1998). Mass Transportation Problems Volume I: Theory, Volume II: Applications.

[28] Rüschendorf L. (2014). Mathematische Statistik.

[29] Ch Pflug G., Pichler A. (2014). Multistage Stochastic Optimization.

